# PERK‐STING‐RIPK3 pathway facilitates cognitive impairment by inducing neuronal necroptosis in sepsis‐associated encephalopathy

**DOI:** 10.1111/cns.14095

**Published:** 2023-01-24

**Authors:** Guo Xiaofeng, Wu You, Jia Qi, Ma Hongwei, Fan Zhongmin, Wang Shiquan, Du Lixia, Peng Yuliang, Fang Zongping, Zhang Xijing

**Affiliations:** ^1^ Department of Critical Care Medicine Xijing Hospital, The Fourth Military Medical University China; ^2^ Department of Intensive Care Unit Joint Logistics Force No. 988 Hospital Zhengzhou China

**Keywords:** necroptosis, PERK, RIPK3, sepsis‐associated encephalopathy, STING

## Abstract

**Aims:**

Sepsis‐associated encephalopathy (SAE) is a common but serious complication in septic survivors and often causes long‐term cognitive impairments. The role of RIPK3‐participated necroptosis in SAE remains obscured. STING is a key molecule in regulating necroptosis and apoptosis. However, there is uncertainty as to the mechanisms of STING in CLP‐induced SAE. The aim of this study was to investigate whether STING is involved in the underlying mechanism of SAE.

**Methods:**

The contextual fear conditioning test (CFCT) assesses cognitive impairment. A transmission electron microscope (TEM) was used to notice the necroptosis. Western blotting and immunofluorescence labeling were applied for the observation of related proteins.

**Results:**

The phosphorylated STING in the hippocampal neuron of SAE mice was significantly elevated. Knocking down STING inhibited necroptosis and attenuated cognitive impairment in SAE mice. Moreover, RIPK3^−/−^ mice had less cognitive deficit in the SAE model. However, STING overexpression did not deteriorate cognitive impairment in RIPK3^−/−^ mice with SAE, indicating that STING is upstream involved in necroptosis. Furthermore, PERK inhibition ameliorated cognitive deficits through a STING‐dependent pathway in SAE mice.

**Conclusion:**

PERK‐STING‐RIPK3 pathway facilitates cognitive impairment by inducing neuronal necroptosis in the pathology of SAE, which provided a new therapeutic target in SAE treatment.

## Graphical abstract

This study is the first to elucidate the role of STING in SAE; additionally, the study demonstrates that the PERK‐STING‐RIPK3 pathway facilitates cognitive impairment by inducing neuronal necroptosis in the pathology of SAE, which provided a new therapeutic target in SAE treatment.

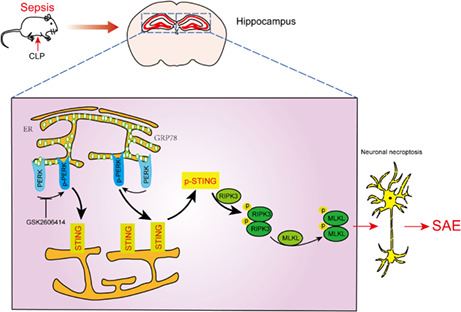

## 1 INTRODUCTION

Sepsis‐associated encephalopathy (SAE) is characterized by no direct infection of the central nervous system (CNS), but diffuse brain dysfunction caused by an infection in the body.^1^ SAE is highly associated with increased morbidity and mortality. Meanwhile, it is reported that SAE survivors acquire long‐term cognitive impairment (10%–20%).^2^, ^3^ Few studies have shown that the pathogenesis of SAE includes disruption of the blood–brain barrier, neuroinflammation, and neuronal death.^4^ Clinically, imaging studies and postmortem examinations of SAE brains showed a reduction in hippocampal volume and cell number. However, the underlying mechanism is far from elucidated.

Necroptosis is a form of programmed cell death (PCD). Emerging evidence has suggested that neuronal necroptosis is associated with neurodegenerative diseases (NDs).^5^ Inhibition of necroptosis in NDs effectively alleviates cognitive impairments.^6^, ^7^, ^8^ However, there is uncertainty regarding the mechanisms of necroptosis in SAE. Understanding the molecular mechanisms underlying the necroptosis process in SAE will be invaluable in developing new therapeutic approaches.

The stimulator of interferon gene (STING; also known as MPYS, TMEM173, and ERIS) is a transmembrane protein,^9^ which is also located in the outer membrane of the endoplasmic reticulum (ER).^10^ Various pathologic conditions can activate ER stress, which plays an important role in cellular homeostasis or cell death programs.^11^ In addition, increasing evidence indicates that STING is involved in regulating various PCD processes in a variety of diseases, ranging from cancer to inflammatory diseases.^12^, ^13^, ^14^ However, whether STING and ER stress are involved in cognitive impairments during SAE through regulating necroptosis needs investigation.

Herein, we tested the hypothesis that protein kinase‐like endoplasmic reticulum kinase (PERK)‐STING‐receptor‐interacting serine/threonine protein kinase 3 (RIPK3) signaling can promote necroptosis and aggravate cognitive dysfunction in SAE mice. We proved that knocking down STING ameliorated hippocampal neuronal necroptosis, thereby alleviating long‐term cognitive dysfunction. Moreover, the overexpression of STING did not deteriorate cognitive dysfunction in SAE RIPK3 knockout mice. Therefore, our results may provide evidence for treatment that may improve prospects against SAE.

## 2 MATERIALS AND METHODS

### 2.1 Animals

The Air Force Medical University (Xi'an, China) provided C57BL/6 (20–25 g) male mice aged 6–8 weeks. The RIPK3^−/−^ mice were gifts from the Basic Medicine College of Air Force Medical University. All animal experiments passed the Air Force Medical University Ethical Inspection.

### 2.2 Cecal ligation and puncture (CLP)

Isoflurane at volumes 20 ml/L and 15 ml/L was used for the induction and maintenance anesthesia stage. Under sterile conditions, the skin and peritoneum were cut, respectively. A 4–0 surgical line was used to ligate the midpoint of the cecum. A 22‐G needle was used to puncture the cecum, and the cecum was gently squeezed to drain the intestinal contents. Next, the peritoneum and skin layers were sutured in turn. To relieve postoperative pain, we choose a subcutaneous injection of 0.1 ml of 0.2% lidocaine. Postoperatively, 0.9% saline (5 ml/kg) was injected subcutaneously.

### 2.3 Open field test (OFT)

Before the experiment, the mice adapted to a quiet environment for 2 h. The test time was between 18:00 and 20:00 h. During each experiment, we placed the mice in the test chamber (40 × 40 × 40 cm) in the same position and direction (Figure S1A). The first 5 min were selected for preadaptation, and the last 5 min comprised of the detection time. The motion track and time course of mice were recorded by an automatic recorder during the detection time. After each mouse was tested, the inner wall and bottom surface of the test chamber were cleaned with 75% alcohol.

### 2.4 Y‐maze test

A Y‐maze made of gray polyvinylidene was placed in a quiet and illuminated room. Each maze comprised three arms (8 × 30 × 15 cm, width×length×height), with an angle of 120 degrees between each arm (Figure S1C). The three arms included the starting arm, wherein the mouse started the exploration; the novel arm, which was blocked at the first trial and was opened at the second trial; and the other. In the experiment, the starting arm and other arm were designed randomly to avoid spatial memory‐related errors. The Y maze test consisted of two trials separated by an intertrial inteval (ITI). The first trial (training; 10 min) allowed the mouse to explore the starting and other arms with the novel arm being blocked. After a 2‐h ITI, the second trial (retention) was conducted, in which the mouse was placed back in the same starting arm with free access to all three arms for 5 min. A video camera linked to the Any‐Maze animal tracking system was installed 60 cm above the chamber to monitor and analyze the number of entries and the time spent in each arm, which was indicative of spatial recognition memory (learned behavior). To remove olfactory cues, each arm of the Y‐maze was cleaned with 75% ethanol solution between trials.

### 2.5 Contextual fear conditioning test

The mice were tested in a contextual fear conditioning paradigm comprising three sessions: habituation, training and retrieval phases (Figure S1B). In the habituation phase, they were adapted to the environment for 2 h and then placed in the training context for 10 min, allowing free exploration. After 24 h, for memory formation, mice were placed in the same context, and 5 s foot shocks (current: 0.6 mA, 2 s; interfoot shock interval: 1 min) was delivered. In the retrieval phase, a fear memory was detected by re‐exposure to the conditioning context in the absence of 5 min shock. The absence of movement other than respiration detected for 2 s was considered to denote freezing, and freezing time was automatically recorded (Xeye Instruments, Beijing MacroAmbition S&T Development Co, Ltd) and expressed as a percentage of time spent freezing.

### 2.6 Nissl staining

The brain slices were stained with 0.1% tar violet for 5 min. Subsequently, they were was washed with PBS. Brain tissue sections were successively dehydrated by 70%, 80%, 95%, 100%, and 100% alcohol gradient, and then washed with xylene. Finally, the brain tissue was sealed with neutral gum. We observed hippocampal CA1 with a microscope and recorded the number of neurons.

### 2.7 Transmission electron microscope

The hippocampal CA1 samples (1 mm^3^) were fixed in 2.5% glutaraldehyde. Subsequently, it fixed with 1% osmic acid fixative. Next, the prepared samples were successively dehydrated with 50%, 70%, 80%, 90%, and 100% alcohol gradients. The samples were then embedded with pure acetone and embedding solution, and cured using an oven. The 3% uranium acetate and lead citrate were used to stain. The cellular structure was photographed with a Hitachi HT7700 transmission electron microscopy (TEM).

### 2.8 Stereotaxic injection

The dorsal hippocampus is the injection target (ML: ±1.60 mm; AP: −2.0 mm; DV: −1.6 mm). The bilateral hippocampus was injected with total of 400 nl of STING‐shRNA, scRNA, or AAV‐hSyn‐STING, AAV‐hSyn (Brain VTA, Wuhan, China). In order to inject the PERK inhibitor GSK2606414 (Cayman, Item No 17376), we placed a cannula in the lateral ventricle (ML: +1.00 mm; AP: −0.50 mm; DV: −1.50 mm) of mice. The 2 μl of GSK2606414 (1 mg/ml) was injected into the lateral ventricle.

### 2.9 Western blot

Samples were dissolved in a lysis buffer (RIPA: phosphatase protease inhibitor: protease inhibitor = 100:1:1). The protein was separated with SDS‐PAGE gel and then transferred to PVDF membranes. The membranes were then incubated overnight at 4°C with the primary antibodies: anti‐p‐STING (ThermoFisher, PA5‐105674, 1:500, rabbit), anti‐STING (GeneTex, GTX33549, 1:1000, rabbit), anti‐p‐RIPK3 (Abcam, ab24182, 1:500, rabbit), anti‐RIPK3 (Abcam, ab62344, rabbit), anti‐p‐MLKL (Abcam, ab196436, 1:500, rabbit), anti‐MLKL (Abcam, Cambridge, ab243142, 1:500, rabbit), anti‐p‐PERK (Cell Signaling, mAb#3179, 1:500, rabbit), anti‐PERK (GeneTex, GTX129275, 1:1000, rabbit), GRP78 (Abcam, ab21685, 1:1000, rabbit), and anti‐GADPH (GeneTex, GTX239 1:1000, mouse). Subsequently, they were incubated for 2 h at room temperature with the HRP‐conjugated secondary antibodies. The target proteins of membranes were detected via enhanced chemiluminescence in ChemiDoc TM imaging equipment (Bio‐Rad, CA, USA).

### 2.10 Immunofluorescence

The brain tissue sections were blocked with mixed liquid (10% donkey serum:0.3% Triton X‐100 = 1:1) for 2 h. Subsequently, they were incubated overnight at 4°C refrigerator with the primary antibodies: anti‐p‐STING (ThermoFisher, PA5‐105674, 1:100, rabbit), anti‐p‐MLKL (Abcam, ab196436, 1:100, rabbit), anti‐p‐RIPK3 (Abcam, ab195117, 1:100, mouse), anti‐p‐PERK (Cell Signaling, mAb#3179, 1:100, rabbit), anti‐Iba1 (Abcam, ab5076, 1:300, goat), anti‐NeuN (Abcam, ab104224, 1:300, mouse), or anti‐GFAP (GeneTex, GTX85454, 1:400, chicken). Subsequently, they were incubated for 2 h at room temperature with the secondary antibodies: Alexa 488‐conjugated donkey‐anti‐mouse IgG (Invitrogen, 1:300) and Alexa 488‐conjugated donkey‐anti‐chicken IgG (Invitrogen, 1:300), Alexa 594‐conjugated donkey‐anti‐rabbit IgG (Invitrogen, 1:300), or Alexa 488–conjugated donkey‐anti‐goat IgG (Invitrogen, 1:300). The tissue sections were observed via a confocal laser scanning microscope (FV10, Olympus, Japan).

### 2.11 Statistical analysis

All data were analyzed with GraphPad Prism 9.0 software. The data from the experiments are expressed as the mean ± SD. To examine the distribution of the data, the Shapiro–Wilk normality test was used. The data (two groups) were analyzed using the student's *t*‐test. The data (more than two groups) were analyzed using one‐way or two‐way ANOVA analysis of variance followed by dunnett's multiple comparisons test or sidak's multiple comparisons test. *p* < 0.05 indicated statistical significance.

## 3 RESULTS

### 3.1 Sepsis‐induced cognitive deficits and upregulated necroptosis in the hippocampus of the mice

**FIGURE 1:**
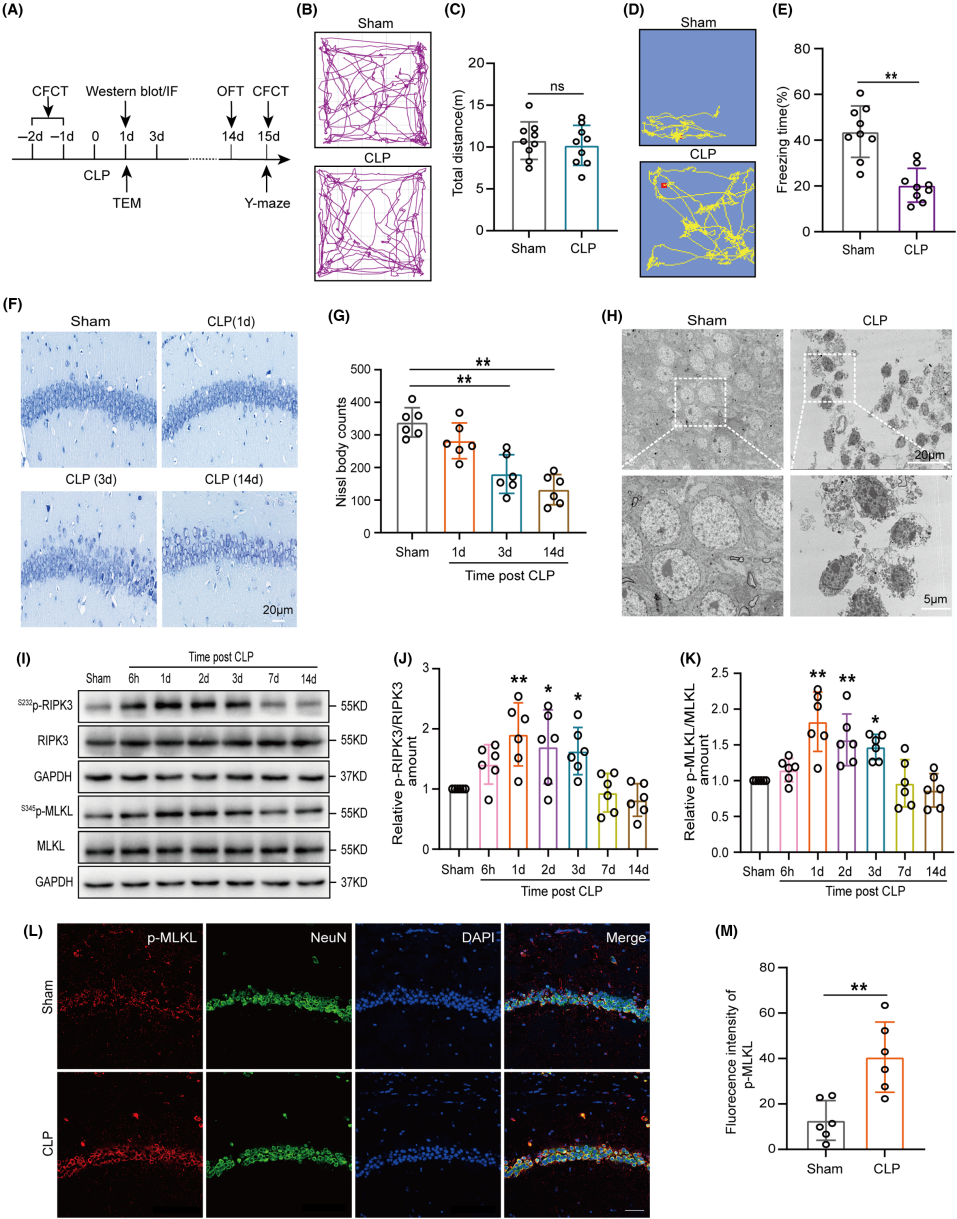
Sepsis‐induced cognitive deficits and upregulated necroptosis in the hippocampus of the mice. (A) Schematic timeline of behavioral test, transmission electron microscopy (TEM), and western blot. (B) Open field test (OFT) trajectory diagram and (C) total distance statistical results (df = 16. *p* = 0.6106, *n* = 9). (D) Contextual fear conditioning test (CFCT) trajectory diagram and (E) freezing time statistical results of CFCT (df = 16. **p < 0.01, *n* = 9). *p* > 0.05, **p < 0.01 versus Sham. The student's *t*‐test was used for statistical analysis. ns: no significant. (F) Nissl staining and (G) Nissl bodies count statistical results in the CA1 region of the hippocampus 1 day, 3 days, and 14 days after CLP (df = 20. **p < 0.01, *n* = 6), scale bar 20 μm. **p < 0.01 versus Sham. One‐way ANOVA with Dunnett's multiple comparisons test was used for statistical analysis. (H) Representative TEM CA1 region of the hippocampus show necroptosis at 1 day after CLP (magnification, 4.9 K and 14 K; Scale bar, 20 μm and 5 μm). (I) Western blot and (J, K) statistical results of western blots of ^S232^p‐RIPK3/RIPK3 (df = 35. **p = 0.0017, 1d versus Sham; *p = 0.0198, 2d versus Sham;*p = 0.0421, 3d versus Sham, *n* = 6) and ^S345^p‐MLKL/MLKL (df = 35. **p < 0.001, 1d versus Sham; **p = 0.0050, 2d versus Sham;*p = 0.0250, 3d versus Sham, *n* = 6). An internal control was GAPDH. One‐way ANOVA with Dunnett's multiple comparisons test was used for statistical analysis. (L) Immunofluorescence staining of p‐MLKL in hippocampus CA1 region 1 day after CLP. Magnification, 40 × Scale bar, 40 μm, and (M) statistical results of fluorescence intensity of p‐MLKL in hippocampus (df = 10. **p = 0.0032, *n* = 6). **p < 0.01 versus Sham. The student's *t*‐test was used for statistical analysis.

In our experiments, after cecal ligation and puncture (CLP), the survival rate of 7‐day is 52.9% (9/17) and the survival rate of 14‐day is 47.05% (8/17) (Figure S1D). First, we used an OFT to evaluate the motor ability of mice 14 days after CLP operation (Figure 1A). The results showed that there was no statistical difference in the total distance of movement between the Sham group and the CLP group (Figures 1B,C), indicating that CLP mice recover normal motor ability. Subsequently, the Y‐maze test and contextual fear conditioning test (CFCT) were used to test whether the memory was impaired in mice 15 days after CLP operation (Figure 1A). The results showed that the number to novel arm (% to total entrance), time to novel arm (% to total time) and freezing time were less in the CLP group compared with the Sham group, suggesting that sepsis induces memory impairment (Figure S1E–H, Figure 1D,E).

In CLP mice, the H&E and Nissl staining indicated that neurons in the hippocampal CA1 region were more damaged at 3 days and 14 days, compared with sham mice (Figure S1I, Figure 1F,G). To further observe the ultrastructure of cells, we observed neurons in the hippocampal CA1 area through TEM. Evidently, we found necroptotic neurons (loss of plasma membrane integrity and nuclear condensation) in CLP mice, whereas cell membrane, organelles, and nuclei were normal in sham mice (Figure 1H). Emerging evidence suggests that necroptosis of neuronal cells is a prominent feature of neurodegenerative disorders and a direct instigator of cognitive decline is unclear. We determine whether there is neuronal necroptosis in the hippocampal tissue of SAE mice. Therefore, we detected necroptosis‐associated proteins (p‐RIPK3, RIPK3, p‐MLKL, and MLKL) in the hippocampal tissue. Surprisingly, our results showed that the expression levels of p‐RIPK3/RIPK3 and p‐MLKL/MLKL were significantly increased in the first 3 days after CLP (Figure 1I–K). Furthermore, immunofluorescence (IF) results indicated increased necroptosis in the CA1 hippocampal neurons 1 day after CLP (Figure 1L,M). Thus, necroptosis was significantly increased in hippocampal neurons of SAE mice.

### 3.2 STING was upregulated in hippocampal tissue of SAE mice

As a key molecule in regulating cell death, the role of STING in SAE is not clear. To demonstrate the activation of STING signaling in the hippocampal tissue of the SAE mice, mice brain tissues were collected at 6 h, 1 day, 2 days, 3 days, 7 days, and 14 days following the surgery.

**FIGURE 2:**
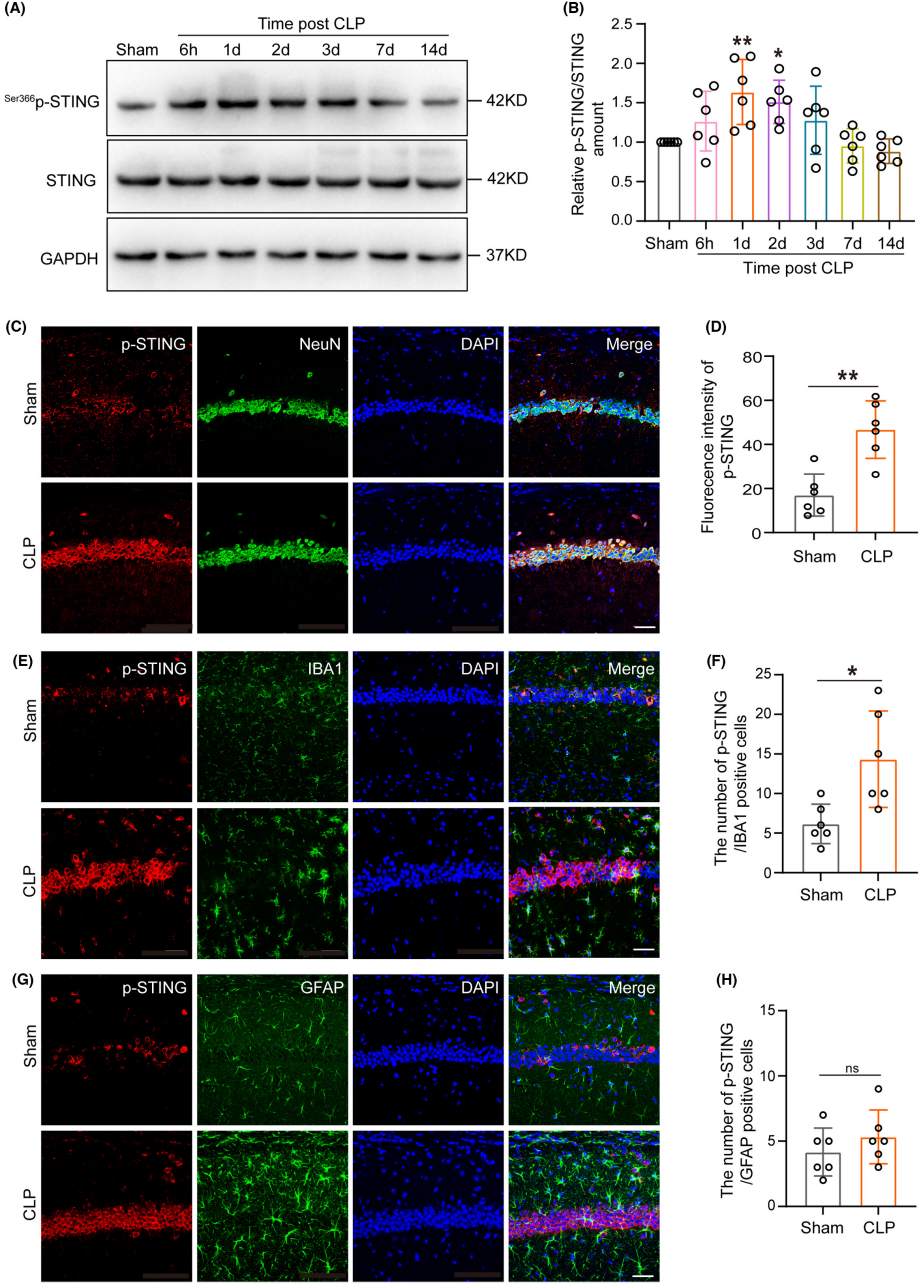
STING was upregulated in hippocampal tissue of SAE mice. (A) Western blot and (B) statistical results of western blots of ^Ser366^p‐STING/STING (df = 35. **p = 0.0047, 1d versus Sham; *p = 0.0293, 2d versus Sham, *n* = 6). An internal control was GAPDH. One‐way ANOVA with Dunnett's multiple comparisons test was used for statistical analysis. (C, E, G) Immunofluorescence staining of p‐STING in neurons, microglia, and astrocyte in hippocampus CA1 region at 1 day after cecal ligation and puncture. Magnification, 40 × Scale bar, 40 μm. (D, F, H) Statistical results of fluorescence intensity of p‐STING or immunofluorescence staining positive cells of p‐STING and microglia, astrocyte in hippocampus (df = 10. **p = 0.0011, *p = 0.0124, *p* = 0.3253, respectively, *n* = 6). *p* > 0.05, *p < 0.05 and **p < 0.01 versus Sham. The student's *t*‐test was used for statistical analysis. ns: no significant.

Our results found that the relative p‐STING/STING amount was significantly increased in the hippocampal tissue 1 and 2 days after CLP (Figure 2A,B). Then, we investigated the distribution of p‐STING in the brain in SAE mice by IF. The results showed in the CA1 region in the hippocampus the p‐STING's expression in neurons was significantly increased (Figure 2C,D), while there was also a small amount of p‐STING expression in microglia and astrocyte (Figure 2E–H). These results mainly indicated that p‐STING in hippocampal CA1 region neurons was increased in SAE mice.

### 3.3 Knocking down STING attenuated cognitive dysfunction and necroptosis in SAE mice

**FIGURE 3:**
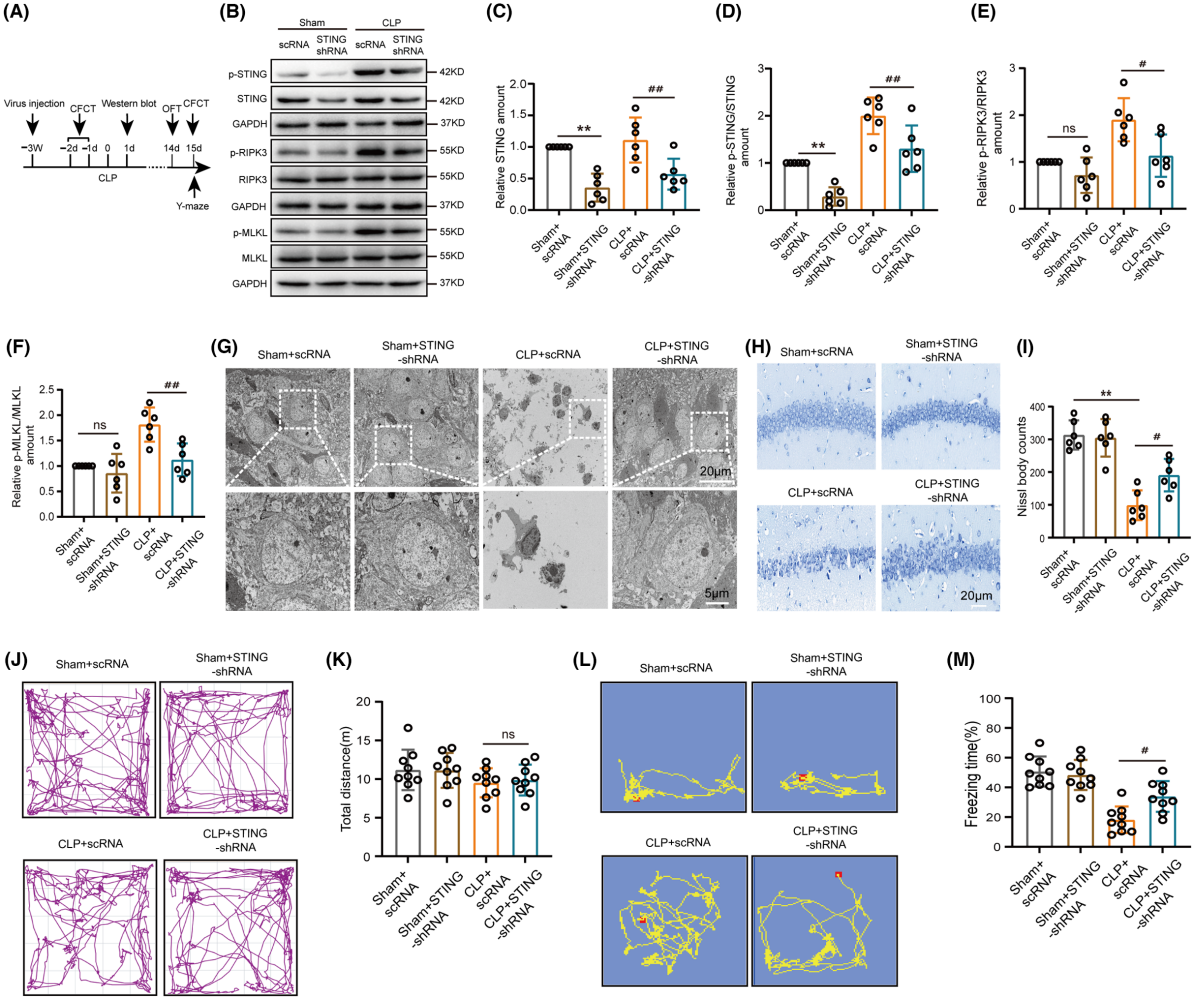
Knocking down STING attenuated cognitive dysfunction and necroptosis in SAE mice. (A) Time diagram of behavior test and virus injection. (B) Western blot and (C–F) statistical results of western blots of STING (df = 20.**p = 0.0011, ^##^ *p* = 0.0060, *n* = 6), p‐STING/STING (df = 20. **p = 0.0077, ^##^ *p* = 0.0089, *n* = 6), p‐RIPK3/RIPK3 (df = 20.*p* = 0.7517, ^#^ *p* = 0.0120, *n* = 6), and p‐MLKL/MLKL (df = 20.*p* = 0.9627, ^##^ *p* = 0.0043, *n* = 6) in hippocampus with virus. An internal control was GAPDH. (G) The TEM of the hippocampal CA1 region show cell morphology at 1 d after CLP (magnification, 4.9 K and 14 K; Scale bar, 20 μm and 5 μm). (H) Nissl staining and (I) Nissl bodies counts statistical results in the CA1 region of the hippocampus with virus 14 days after Sham+CLP (df = 20. **p < 0.01, ^#^ *p* = 0.0254, *n* = 6), scale bar 20 μm. (J) Open field test (OFT) trajectory diagram and (K) total traveling distance statistical results of OFT (df = 32.*p* = 0.9997, *n* = 9). (L) Contextual fear conditioning test (CFCT) and (M) freezing time statistical results of CFCT (df = 32. ^#^ *p* = 0.0123, *n* = 9). *p* > 0.05, **p < 0.01 versus Sham+scRNA, *p* > 0.05, ^#^ *p* < 0.05, ^##^ *p* < 0.01 versus CLP + scRNA. Two‐way ANOVA with Sidak's multiple comparisons test was used for statistical analysis. ns: no significant.

To test the essential role of STING in cognitive deficits and necroptosis in SAE mice, we conducted STING‐shRNA to knockdown STING expression. Using intrahippocampal injection, we transfected AAV particles expressing a STING‐shRNA and scRNA into hippocampal of six‐week‐old C57BL/6J mice (Figure S2A,B). After 3 weeks, these mice underwent sham and CLP surgery (Figure 3A). First, we verified the effectiveness of STING‐shRNA knockdown STING expression. Our results found that the relative STING or p‐STING/STING amount were downregulated in the mice's hippocampus with STING‐shRNA (Figure 3B–D). Meanwhile, the results also showed that STING‐shRNA significantly inhibited p‐RIPK3/RIPK3, p‐MLKL/MLKL expression levels in CLP mice compared with scRNA (Figures 3B,E,F). Moreover, TEM demonstrated more necroptosis in the hippocampal CA1 region from CLP + scRNA mice than the CLP + STING‐shRNA mice (Figure 3G). The H&E and Nissl staining results showed that the neuron counts in the CA1 region of hippocampus in the CLP + STING‐shRNA mice were meaningfully ameliorated compared with CLP + scRNA mice (Figure S2G, Figure 3H,I). Next, OFT indicated that the total distance of mice between the four groups was not significant (Figure 3J,K). The Y‐maze test and CFCT were used to test whether STING‐shRNA could ameliorate the cognitive deficit of SAE mice. As predicted, CLP + STING‐shRNA mice significantly increased number to novel arm (% to total entrance), time to novel arm (% to total time) and freezing time compared with the CLP + scRNA mice (Figure S2C–F, Figure 3L,M), demonstrating that STING knockdown can improved their cognitive ability when subjected to CLP. These results suggested that knockdown STING in the hippocampal neurons could reduce neuronal necroptosis and alleviate cognitive impairments in SAE mice.

### 3.4 Overexpression of STING deteriorated cognitive dysfunction in SAE mice in a RIPK3 dependent manner

**FIGURE 4:**
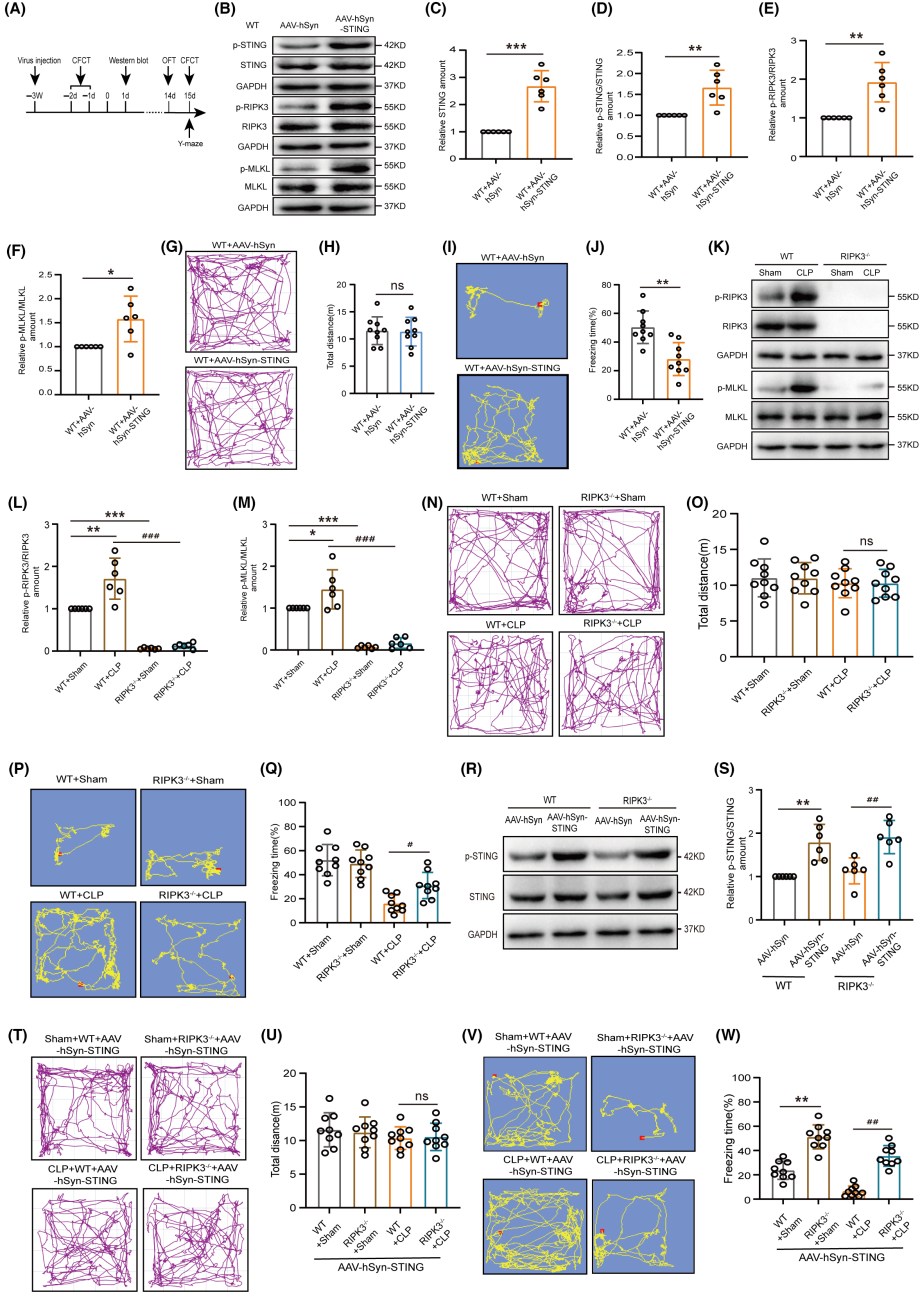
Overexpression of STING deteriorated cognitive dysfunction in SAE mice in a RIPK3 dependent manner. (A) Time diagram of behavior test and virus injection. (B) Western blot and (C–F) statistical results of western blots of STING (df = 10. ***p < 0.001, *n* = 6), p‐STING/STING (df = 10. **p = 0.0030, *n* = 6), p‐RIPK3/RIPK3 (df = 10. **p = 0.0013, *n* = 6), and p‐MLKL/MLKL (df = 10. *p = 0.0139, *n* = 6) in hippocampus 3 weeks after the virus injection. An internal control was GAPDH. *p < 0.05, **p < 0.01, ***p < 0.001 versus WT + AAV‐hSyn. The student's *t*‐test was used for statistical analysis. (G, N, T) Open field test (OFT) trajectory diagram and (H, O, U) total traveling distance statistical results of OFT (df = 16. *p* = 0.8838; df = 32. *p* > 0.9999; df = 32. *p* > 0.9999, respectively, *n* = 9). (I) Contextual fear conditioning test (CFCT) and (J) freezing time statistical results of CFCT (df = 16. **p < 0.01, *n* = 9). *p* > 0.05, **p < 0.01 versus WT + AAV‐hSyn. The student's *t*‐test was used for statistical analysis. (K) Western blot and (L, M) statistical results of western blots of hippocampal p‐RIPK3/RIPK3 (df = 20. **p < 0.01, ^###^ *p* < 0.001, *n* = 6), and p‐MLKL/MLKL (df = 20. *p = 0.0274, ^###^ *p* < 0.001, *n* = 6) in wild‐type (WT) and RIPK3^−/−^ mice. An internal control was GAPDH. (P) CFCT and (Q) freezing time statistical results of CFCT about WT and RIPK3^−/−^ mice (df = 32. ^#^ *p* = 0.0360, *n* = 9). *p < 0.05, **p < 0.01, ***p < 0.001 versus WT + sham; *p* > 0.05, ^#^ *p* < 0.05, ^###^ *p* < 0.001 versus WT + CLP. Two‐way ANOVA with Sidak's multiple comparisons test was used for statistical analysis. (R) Western blot and (S) statistical results of hippocampal p‐STING/STING (df = 20. **p = 0.0022, ^##^ *p* = 0.0025, *n* = 6) expression in the WT and RIPK3^−/−^ mice with virus. An internal control was GAPDH. **p < 0.01 versus WT + AAV‐hSyn, ^##^ *p* < 0.01 versus RIPK3^−/−^+ AAV‐hSyn. Two‐way ANOVA with Sidak's multiple comparisons test was used for statistical analysis. (V) CFCT and (W) freezing time statistical results of CFCT about the WT and RIPK3^−/−^ mice with upregulation of STING virus (df = 32. **p < 0.01, ^##^ *p* < 0.01, *n* = 9). **p < 0.01 versus WT + sham+AAV‐hSyn‐STING, *p* > 0.05, ^##^ *p* < 0.01 versus WT + CLP + AAV‐hSyn‐STING. Two‐way ANOVA with Sidak's multiple comparisons test was used for statistical analysis. ns: no significant.

To corroborated the role of STING in mediating the pathologic process of SAE, we employed intrahippocampal injection and conducted AAV‐hSyn‐STING or AAV‐hSyn (Figure S3A,B, Figure 4A). The western blot assay showed that STING and p‐STING/STING expression were meaningfully upregulated in the hippocampal tissue of mice with AAV‐hSyn‐STING (Figure 4B–D). It also showed that AAV‐hSyn‐STING significantly upregulated p‐RIPK3/p‐RIPK3 and p‐MLKL/MLKL (Figure 4B,E,F). Next, we used an OFT to test general locomotor activities. Notably, there was no statistical difference in total distance between the two groups (Figure 4G,H). The Y‐maze test and CFCT were performed to explore whether overexpression of STING could impair the cognitive function of mice. As predicted, the AAV‐hSyn‐STING mice showed a less number to novel arm (% to total entrance), time to novel arm (% to total time) and freezing time than the AAV‐hSyn mice (Figure S3C–F, Figure 4I,J), demonstrating that overexpression of STING impaired the cognitive function of mice.

Next, RIPK3^−/−^ mice were examined in our study. The western blot assay showed that RIPK3 knockout mice rarely expressed p‐RIPK3 and p‐MLKL proteins (Figure 4K–M). OFT indicated that total distances of sham and CLP groups were not different after 14 days of surgery (Figure 4N,O). The Y‐maze test and CFCT showed that RIPK3^−/−^ mice treated with CLP indicated more improvement number to novel arm (% to total entrance), time to novel arm (% to total time) and freezing time than wild‐type (WT) mice of CLP (Figure S3G–J, Figure 4P,Q), indicating an important role of necroptotic signaling in cognitive function.

We hypothesized that elevated STING signaling in RIPK3^−/−^ mice that underwent CLP may not deteriorate cognitive dysfunction. To test this, we upregulated hippocampal STING expression in six‐week‐old RIPK3^−/−^ mice by bilateral injection of AAV‐hSyn‐STING. To test the effectiveness of AAV‐hSyn‐STING in regulating STING overexpression we performed a western blot assay. The expression of STING was significantly upregulated in the hippocampus with AAV‐hSyn‐STING (Figure 4R,S). Next, OFT indicated that total distance between the four groups was not significant (Figure 4T,U). The Y‐maze test and CFCT was used to test whether AAV‐hSyn‐STING could deteriorate cognitive dysfunction in RIPK3^−/−^ mice that suffered CLP. Notably, they had a more improvement number to novel arm (% to total entrance), time to novel arm (% to total time), and freezing time compared with the WT mice with CLP (Figure S3K–N, Figure 4V,W), demonstrating that AAV‐hSyn‐STING could not deteriorate cognitive dysfunction.

### 3.5 Inhibition of PERK activity ameliorated cognitive deficits via STING pathway in SAE mice

**FIGURE 5:**
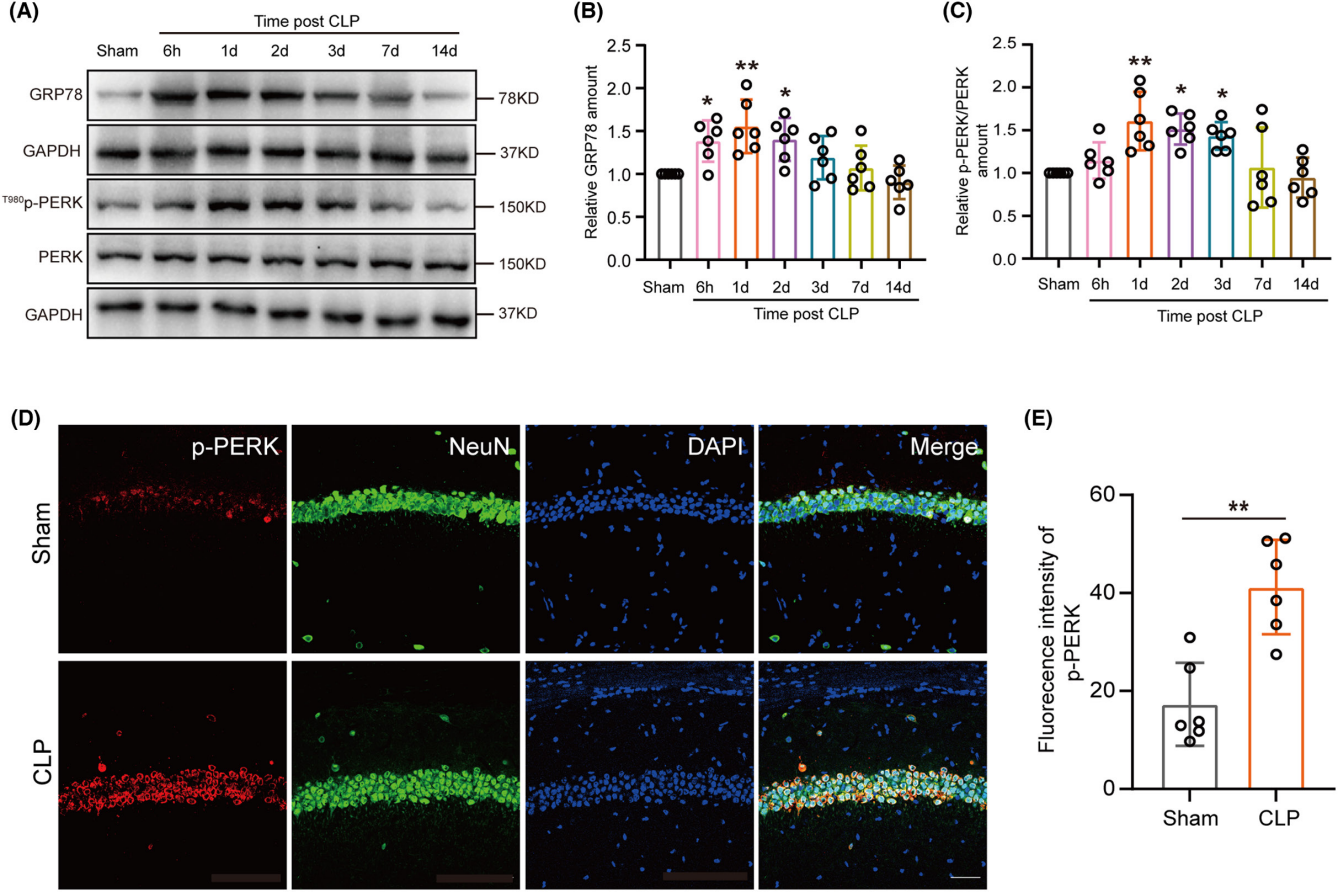
Endoplasmic reticulum stress was induced in hippocampal tissue of SAE mice. (A) Western blot and (B, C) western blot analysis for GRP78 (df = 35. *p = 0.0372, 6 h versus sham; **p = 0.0014, 1d versus sham; *p = 0.0267, 2d versus sham, *n* = 6) and ^T980^p‐PERK/PERK (df = 35. **p = 0.0019, 1d versus sham; *p = 0.0100, 2d versus sham; *p = 0.0370, 3d versus sham, *n* = 6) expression in hippocampus after CLP surgery. An internal control was GAPDH. One‐way ANOVA with Dunnett's multiple comparisons test was used for statistical analysis. (D) Immunofluorescence staining of p‐PERK in the hippocampus CA1 region neurons 1 day after CLP. Magnification, 40 × Scale bar, 40 μm and (E) statistical results of fluorescence intensity of p‐PERK in hippocampus (df = 10. **p = 0.0010, *n* = 6). **p < 0.01 versus Sham. The student's *t*‐test was used for statistical analysis.

**FIGURE 6:**
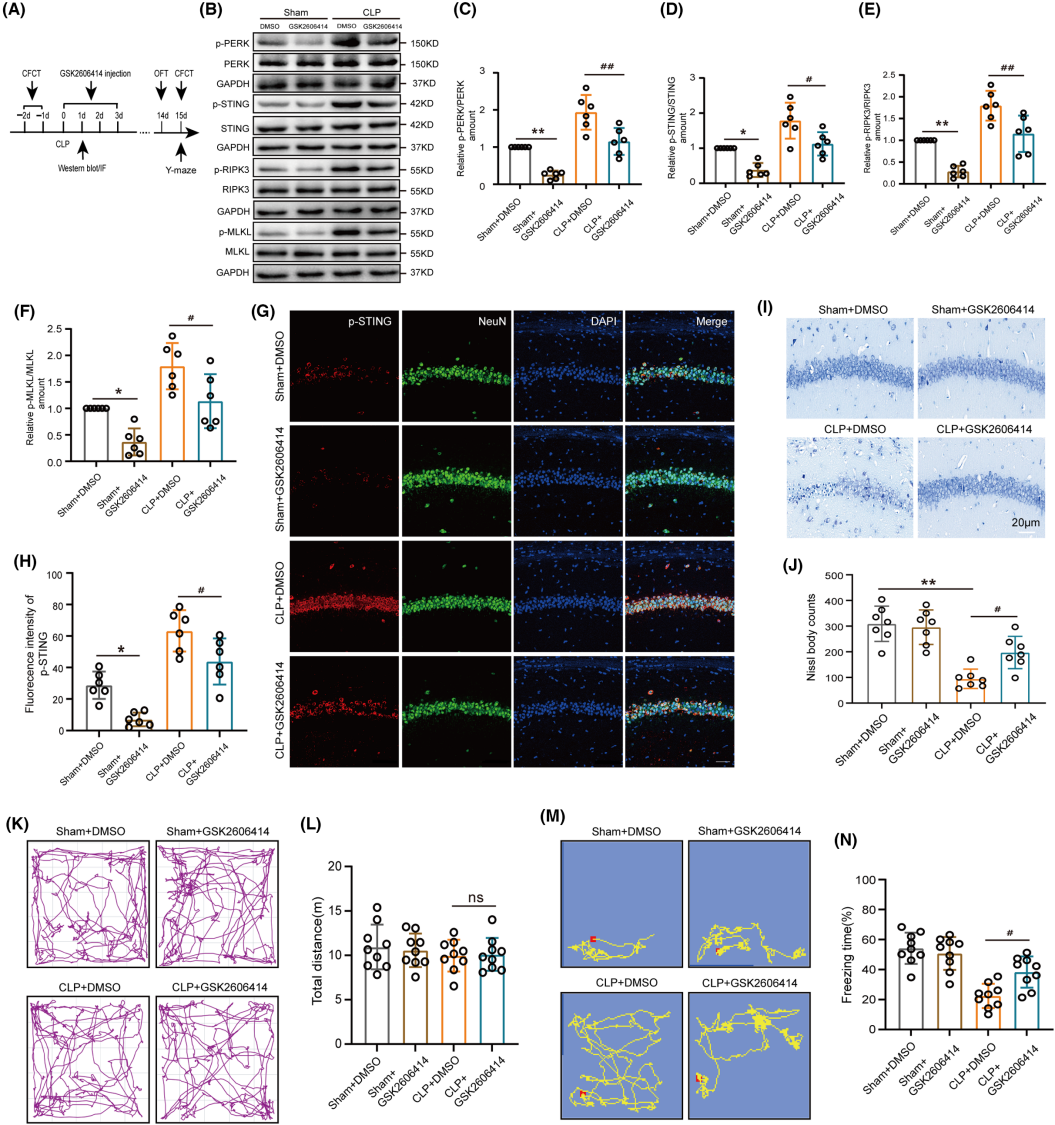
Inhibition of PERK activity ameliorated cognitive deficits via STING pathway in SAE mice. (A) Time diagram of PERK inhibitor GSK2606414 injection and behavioral test. (B) Western blot and (C–F) statistical results of hippocampal expression of p‐PERK/PERK (df = 20. **p = 0.0021, ^##^ *p* = 0.0013, *n* = 6), p‐STING/STING (df = 20. *p = 0.0189, ^#^ *p* = 0.0115, *n* = 6), p‐RIPK3/RIPK3 (df = 20. **p = 0.0014, ^##^ *p* = 0.0041, *n* = 6), and p‐MLKL/MLKL (df = 20. *p = 0.0372, ^#^ *p* = 0.0273, *n* = 6) after GSK2606414 inhibitor. An internal control was GAPDH. (G) Immunofluorescence staining of p‐STING after Sham and CLP surgery with or without GSK2606414 treatment. Magnification, 40 × Scale bar, 40 μm. (H) Statistical results of fluorescence intensity of p‐STING in hippocampus (df = 20. *p = 0.0159, ^#^ *p* = 0.0365, *n* = 6). (I) Nissl staining and (J) Nissl bodies count statistical results in the CA1 region of the hippocampus with DMSO or GSK2606414 14 days after Sham+CLP (df = 24.**p < 0.01, ^#^ *p* = 0.0245, *n* = 7), scale bar 20 μm. (K) Open field test (OFT) trajectory diagram and (L) total traveling distance statistical results of OFT (df = 32. *p* > 0.9999, *n* = 9). (M) Contextual fear conditioning test (CFCT) and (N) freezing time statistical results of CFCT (df = 32. ^#^ *p* = 0.0120, *n* = 9).*p < 0.05, **p < 0.01 versus sham+DMSO, *p* > 0.05, ^#^ *p* < 0.05, ^##^ *p* < 0.01 versus CLP + DMSO. Two‐way ANOVA with Sidak's multiple comparisons test was used for statistical analysis. ns: no significant.

To explore the upstream of the STING‐RIPK3 pathway, we looked for the expression of the markers of ER stress. There was a significant increase in glucose‐regulated protein 78 (GRP78) and p‐PERK/PERK after CLP, peaking at 1 day (Figure 5A–C). Immunofluorescence also showed that p‐PERK expression was significantly increased in the neurons of the hippocampal CA1 region of CLP mice compared to sham (Figure 5D,E). To explore the role of PERK in cognitive deficits in SAE mice, the PERK inhibitor GSK2606414 was used in our study (Figure S4A,B, Figure 6A). First, our results indicated that the protein expression of p‐PERK/PERK, p‐STING/STING, p‐RIPK3/RIPK3, and p‐MLKL/MLKL had declined in these hippocampus from PERK inhibitor GSK2606414 mice compared to CLP‐DMSO mice (Figure 6B–F). Furthermore, IF indicated that p‐STING's expression in CA1‐region neurons of the hippocampus was reduced with the treatment of GSK2606414 after CLP (Figure 6G,H). Next, the results of H&E and Nissl staining show decreased neuron counts in the hippocampal CA1 regions of CLP‐DMSO mice. However, this was significantly improved by treatment with GSK2606414 after CLP (Figure S4G, Figure 6I,J). Moreover, an OFT evaluated general locomotor activities. Notably, there was no significance in the total distance among the four groups (Figure 6K,L). The Y‐maze test and CFCT was searched to understand whether PERK inhibitor GSK2606414 could ameliorate to the cognitive function of mice. Notably, CLP mice treated with GSK2606414 showed a more improvement number to novel arm (% to total entrance), time to novel arm (% to total time) and freezing time compared to CLP‐DMSO mice, suggesting that inhibition of PERK by GSK2606414 improves cognitive behavior following CLP (Figure S4C–F, Figure 6M,N).

## 4 DISCUSSION

SAE occurs in approximately 70% of sepsis patients.^15^ Surviving patients with SAE often experience permanent cognitive impairment, including decreased concentration, learning capacity, and memory,^16^ which is closely associated with hippocampal injury.^17^ A recent study has verified that cognitive impairment is caused by hippocampal injury in SAE.^18^ The previous research demonstrated that the CAl pyramidal subfield of the hippocampus is particularly vulnerable to damage.^19^ Meanwhile, neurons in the CA1 area are closely related to learning and memory. In our study, we focused on learning and memory changes in SAE mice. Correspondingly, our Y‐maze test and CFCT results found that number to novel arm (% to total entrance), time to novel arm (% to total time), and freezing time in behavioral memory decreased in CLP mice 15 days after training, indicating hippocampal CA1 area‐dependent memory impairment in the CLP mouse model.^20^ It is reported that the extracellular volume fraction (EVF) of the hippocampal CAl pyramidal subregion is exceptionally low, and is particularly vulnerable to damage.^21^ Therefore, we chose the hippocampal CA1 region as the main observation area. Furthermore, Nissl staining and H&E staining indicated significant neuronal death in the hippocampal CA1 area after CLP.

Emerging evidence has suggested that necroptosis is a newly discovered form of PCD in NDs.^5^, ^21^, ^22^ Necroptosis includes receptor‐interacting serine/threonine protein kinase 1 (RIPK1), RIPK3, and mixed lineage kinase domain‐like pseudo‐kinase (MLKL). Three proteins are sequentially phosphorylated and form necrosomes.^23^Subsequently, the MLKL binds to the cell membrane, causing a hole in the cell membrane, thereby triggering necroptosis.^24^ Accumulating evidence suggests that cognitive impairments are associated with neuronal necroptosis in NDs, such as Parkinson disease (PD) and Alzheimer disease (AD),^21^, ^25^, ^26^ specifically neuronal loss in the hippocampal CA1 regions.^27^ In our study, protein expression levels of p‐RIPK3/RIPK3 and p‐MLKL/MLKL were meaningfully increased in hippocampal tissue of SAE mice. Remarkably, our immunofluorescence images study further showed that p‐MLKL is expressed in hippocampal CA1 neurons in SAE mice. These results suggest that neurons in the hippocampal CA1 regions undergo necroptosis in SAE mice. Research reports have shown that inhibition of necroptosis in mouse models of AD effectively alleviated cognitive impairments.^28^, ^29^ Interestingly, our data suggested that RIPK3 knockout also ameliorates cognitive dysfunction in SAE mice, indicating that necroptosis is associated with cognitive impairment in SAE mice. Therefore, we speculated that necroptosis is highly correlated to hippocampal neuronal death and cognitive impairment in SAE mice. However, how necroptosis is activated in SAE remains unclear.

STING is mainly located in the ER; it consists of 378 amino acids in murine cells.^30^ STING has a C‐terminal domain, which has the ability to recruit and activate TBK1 signaling pathways.^31^, ^32^ Increasing evidence indicates that STING participates in regulating various PCD processes. In LPS‐induced acute lung injury, STING regulates the pyroptosis of lung tissue and macrophages in mice, whereas STING deficiency eliminated the activation of pyroptosis.^33^ Additionally, STING‐IRF3 regulates the apoptosis and inflammation of cardiomyocytes via activation of NLRP3 in LPS‐induced cardiac dysfunction.^34^ Therefore, we hypothesized that neuronal necroptosis was induced by activation of STING. First, we verified the expression of STING in neurons during SAE. Our results showed that p‐STING/STING protein in the hippocampal tissue was increased in SAE mice and that p‐STING was mainly co‐localized with neurons.^35^ Therefore, we found that p‐STING/STING protein is highly associated with pathological process of SAE. Accumulating evidence suggested that STING knockdown relieved cognitive impairment and hippocampal neurogenesis in NDs.^36^, ^37^, ^38^ Accordingly, our results demonstrated that knockdown neuronal STING in CA1 regions of the hippocampus reduced necroptosis and alleviated cognitive impairment in SAE mice. Second, we examined whether STING affected cognitive function by regulating necroptosis in SAE. We found that RIPK3^−/−^ mice had improved cognition over WT mice after CLP surgery. There are reports that administration of STING agonists aggravates cognitive impairment in PD mice.^37^ Our results detected that upregulation neuronal STING in CA1 regions of the hippocampus triggered cognitive impairment in WT mice. Moreover, overexpression of STING did not deteriorate cognitive impairment in SAE RIPK3^−/−^ mice. Therefore, we speculate that STING‐RIPK3 signaling may lead to cognitive impairment in SAE mice.

Research has shown that STING is located in the outer membrane of the ER. The ER is associated with maintaining protein homeostasis and is significantly sensitive to changes in the cellular microenvironment.^39^ Under various stress, ER produces ER stress. The activating transcription factor 6 (ATF6), inositol requiring kinase 1 (IRE1), and PERK are the three pathways of unfolded protein response under ER stress. Research has shown that mild ER stress protects neurons by regulating autophagy,^40^ but persistent or excessive ER stress can cause cell death.^41^ PERK is a central ER stress factor and restores protein homeostasis by inhibiting protein translation.^11^ In our study, we found that GRP78 and p‐PERK/PERK protein in the hippocampal tissue were increased in SAE mice. A recent study has indicated that PERK can interact with STING^42^; therefore, neuronal necroptosis may be regulated by the PERK‐STING axis in SAE. Our results showed that inhibition of PERK declined the expression of p‐STING/STING and p‐RIPK3/RIPK3 in hippocampal tissue with SAE mice. Additionally, IF also showed that PERK inhibitors can reduce the co‐localization of p‐STING with hippocampal CA1 neurons, suggesting that it facilitated neuronal survival. Meanwhile, PERK inhibition also alleviated memory impairment in SAE mice. Therefore, inhibition of the PERK‐STING‐RIPK3 pathway has a neuroprotective effect in SAE mice.

There are some limitations to this study. First, we did not explore the role of necroptosis in STING knockout mice, which warrants further exploration. Second, under severe ER stress conditions, the GRP78 protein is separated from PERK, ATF6, and IRE1 when misfolded proteins accumulate to a certain extent in the ER lumen. Here, only PERK pathway's effects were evaluated, and so the two other transmembrane protein receptors IRE1 and ATF6 require further investigation.

## 5 CONCLUSION

In conclusion, PERK‐STING‐RIPK3 pathway facilitates cognitive impairment by inducing neuronal necroptosis in the pathology of SAE, which provided a new therapeutic target in SAE treatment. Although we used knockdown and overexpression viruses to regulate STING and PERK to alleviate the pathogenesis of SAE, the clinical use of virus intervention has considerable limitations. However, it has been reported that STING and PERK have corresponding inhibitors, which have considerable clinical transfer potential.^43^, ^44^ Furthermore, optimizing the characteristics of these two inhibitors before clinical use needs further exploration.

## AUTHOR CONTRIBUTIONS

XFG, YW, and QJ initiated the research and wrote the manuscript. ZMF and SQW conducted the CLP model. LXD, YLP, and HWM analyzed the data and performed the experiments. ZPF and XJZ revised the manuscript.

## CONFLICT OF INTEREST

The authors declare that they have no potential competing interests.

## Supporting information


Figure S1

Figure S2

Figure S3

Figure S4

## Data Availability

Data relevant to support the current study can be obtained from our corresponding authors.

## References

[1] Gofton TE , Young GB . Sepsis‐associated encephalopathy. Nat Rev Neurol. 2012;8(10):557‐566. 22986430 10.1038/nrneurol.2012.183

[2] Helbing DL , Böhm L , Witte OW . Sepsis‐associated encephalopathy. CMAJ. 2018;190(36):E1083. 30201616 10.1503/cmaj.180454 PMC6131085

[3] Andonegui G , Zelinski EL , Schubert CL , et al. Targeting inflammatory monocytes in sepsis‐associated encephalopathy and long‐term cognitive impairment. JCI Insight. 2018;3(9):e99364. 29720578 10.1172/jci.insight.99364 PMC6012517

[4] Tauber SC , Djukic M , Gossner J , Eiffert H , Brück W , Nau R . Sepsis‐associated encephalopathy and septic encephalitis: an update. Expert Rev Anti Infect Ther. 2021;19(2):215‐231. 32808580 10.1080/14787210.2020.1812384

[5] Zhang S , Tang MB , Luo HY , Shi CH , Xu YM . Necroptosis in neurodegenerative diseases: a potential therapeutic target. Cell Death Dis. 2017;8(6):e2905. 28661482 10.1038/cddis.2017.286 PMC5520937

[6] Qinli Z , Meiqing L , Xia J , et al. Necrostatin‐1 inhibits the degeneration of neural cells induced by aluminum exposure. Restor Neurol Neurosci. 2013;31(5):543‐555. 23735313 10.3233/RNN-120304

[7] Iannielli A , Bido S , Folladori L , et al. Pharmacological inhibition of necroptosis protects from dopaminergic neuronal cell death in Parkinson's disease models. Cell Rep. 2018;22(8):2066‐2079. 29466734 10.1016/j.celrep.2018.01.089 PMC5842028

[8] Nasseri B , Zareian P , Alizade H . Apelin attenuates streptozotocin‐induced learning and memory impairment by modulating necroptosis signaling pathway. Int Immunopharmacol. 2020;84:106546. 32413735 10.1016/j.intimp.2020.106546

[9] Zhang R , Kang R , Tang D . The STING1 network regulates autophagy and cell death. Signal Transduct Target Ther. 2021;6(1):208. 34078874 10.1038/s41392-021-00613-4 PMC8172903

[10] Galluzzi L , Vanpouille‐Box C , Bakhoum SF , Demaria S . SnapShot: CGAS‐STING signaling. Cell. 2018;173(1):276‐276.e1. 29570996 10.1016/j.cell.2018.03.015

[11] Meng C , Zhang J , Dang B , et al. PERK pathway activation promotes intracerebral hemorrhage induced secondary brain injury by inducing neuronal apoptosis both in vivo and in vitro. Front Neurosci. 2018;12:111. 29541018 10.3389/fnins.2018.00111 PMC5835756

[12] Tang D , Kang R , Berghe TV , Vandenabeele P , Kroemer G . The molecular machinery of regulated cell death. Cell Res. 2019;29(5):347‐364. 30948788 10.1038/s41422-019-0164-5 PMC6796845

[13] Barber GN . STING: infection, inflammation and cancer. Nat Rev Immunol. 2015;15(12):760‐770. 26603901 10.1038/nri3921 PMC5004891

[14] Jiang GL , Yang XL , Zhou HJ , et al. cGAS knockdown promotes microglial M2 polarization to alleviate neuroinflammation by inhibiting cGAS‐STING signaling pathway in cerebral ischemic stroke. Brain Res Bull. 2021;171:183‐195. 33745949 10.1016/j.brainresbull.2021.03.010

[15] Catarina AV , Branchini G , Bettoni L , De Oliveira JR , Nunes FB . Sepsis‐associated encephalopathy: from pathophysiology to progress in experimental studies. Mol Neurobiol. 2021;58(6):2770‐2779. 33495934 10.1007/s12035-021-02303-2

[16] Widmann CN , Heneka MT . Long‐term cerebral consequences of sepsis. Lancet Neurol. 2014;13(6):630‐636. 24849863 10.1016/S1474-4422(14)70017-1

[17] Mancini A , de Iure A , Picconi B . Basic mechanisms of plasticity and learning. Handb Clin Neurol. 2022;184:21‐34. 35034736 10.1016/B978-0-12-819410-2.00002-3

[18] Shen Y , Zhang Y , Du J , et al. CXCR5 down‐regulation alleviates cognitive dysfunction in a mouse model of sepsis‐associated encephalopathy: potential role of microglial autophagy and the p38MAPK/NF‐kappaB/STAT3 signaling pathway. J Neuroinflammation. 2021;18(1):246. 34711216 10.1186/s12974-021-02300-1 PMC8554863

[19] McBain CJ , Traynelis SF , Dingledine R . Regional variation of extracellular space in the hippocampus. Science. 1990;249(4969):674‐677. 2382142 10.1126/science.2382142

[20] Fu Q , Wu J , Zhou XY , et al. NLRP3/Caspase‐1 pathway‐induced pyroptosis mediated cognitive deficits in a mouse model of sepsis‐associated encephalopathy. Inflammation. 2019;42(1):306‐318. 30276509 10.1007/s10753-018-0894-4 PMC6394578

[21] Oñate M , Catenaccio A , Salvadores N , et al. The necroptosis machinery mediates axonal degeneration in a model of Parkinson disease. Cell Death Differ. 2020;27(4):1169‐1185. 31591470 10.1038/s41418-019-0408-4 PMC7205895

[22] Ofengeim D , Ito Y , Najafov A , et al. Activation of necroptosis in multiple sclerosis. Cell Rep. 2015;10(11):1836‐1849. 25801023 10.1016/j.celrep.2015.02.051 PMC4494996

[23] Yuan J , Amin P , Ofengeim D . Necroptosis and RIPK1‐mediated neuroinflammation in CNS diseases. Nat Rev Neurosci. 2019;20(1):19‐33. 30467385 10.1038/s41583-018-0093-1 PMC6342007

[24] Grootjans S , Vanden BT , Vandenabeele P . Initiation and execution mechanisms of necroptosis: an overview. Cell Death Differ. 2017;24(7):1184‐1195. 28498367 10.1038/cdd.2017.65 PMC5520172

[25] Park J , Ha HJ , Chung ES , et al. O‐GlcNAcylation ameliorates the pathological manifestations of Alzheimer's disease by inhibiting necroptosis. Sci Adv. 2021;7(3):eabd3207. 33523877 10.1126/sciadv.abd3207 PMC7806231

[26] Mitroshina EV , Loginova MM , Yarkov RS , et al. Inhibition of neuronal necroptosis mediated by RIPK1 provides neuroprotective effects on hypoxia and ischemia in vitro and in vivo. Int J Mol Sci. 2022;23(2):735. 35054920 10.3390/ijms23020735 PMC8775468

[27] Jayaraman A , Htike TT , James R , Picon C , Reynolds R . TNF‐mediated neuroinflammation is linked to neuronal necroptosis in Alzheimer's disease hippocampus. Acta Neuropathol Commun. 2021;9:159. 34625123 10.1186/s40478-021-01264-w PMC8501605

[28] Rubinsztein DC . RIPK1 promotes inflammation and beta‐amyloid accumulation in Alzheimer's disease. Proc Natl Acad Sci U S A. 2017;114(41):10813‐10814. 28973950 10.1073/pnas.1715241114 PMC5642738

[29] Yang SH , Lee DK , Shin J , et al. Nec‐1 alleviates cognitive impairment with reduction of Abeta and tau abnormalities in APP/PS1 mice. EMBO mol Med. 2017;9:61‐77. 27861127 10.15252/emmm.201606566 PMC5210088

[30] Ishikawa H , Barber GN . STING is an endoplasmic reticulum adaptor that facilitates innate immune signalling. Nature. 2008;455(7213):674‐678. 18724357 10.1038/nature07317 PMC2804933

[31] Ouyang S , Song X , Wang Y , et al. Structural analysis of the STING adaptor protein reveals a hydrophobic dimer interface and mode of cyclic di‐GMP binding. Immunity. 2012;36(6):1073‐1086. 22579474 10.1016/j.immuni.2012.03.019 PMC3654694

[32] Gao P , Ascano M , Zillinger T , et al. Structure‐function analysis of STING activation by c[G(2′,5′)pA(3′,5′)p] and targeting by antiviral DMXAA. Cell. 2013;154(4):748‐762. 23910378 10.1016/j.cell.2013.07.023 PMC4386733

[33] Ning L , Wei W , Wenyang J , Rui X , Qing G . Cytosolic DNA‐STING‐NLRP3 axis is involved in murine acute lung injury induced by lipopolysaccharide. Clin Transl Med. 2020;10(7):e228. 33252860 10.1002/ctm2.228 PMC7668192

[34] Li N , Zhou H , Wu H , et al. STING‐IRF3 contributes to lipopolysaccharide‐induced cardiac dysfunction, inflammation, apoptosis and pyroptosis by activating NLRP3. Redox Biol. 2019;24:101215. 31121492 10.1016/j.redox.2019.101215 PMC6529775

[35] Chin AC . PERK‐STING signaling drives neuroinflammation in traumatic brain injury. J Neurosci. 2020;40(12):2384‐2386. 32188742 10.1523/JNEUROSCI.2881-19.2020 PMC7083527

[36] Hou Y , Wei Y , Lautrup S , et al. NAD^+^ supplementation reduces neuroinflammation and cell senescence in a transgenic mouse model of Alzheimer's disease via cGAS–STING. Proc Natl Acad Sci U S A. 2021;118(37):e2011226118. 34497121 10.1073/pnas.2011226118 PMC8449423

[37] Zhao M , Wang B , Zhang C , et al. The DJ1‐Nrf2‐STING axis mediates the neuroprotective effects of withaferin a in Parkinson's disease. Cell Death Differ. 2021;28(8):2517‐2535. 33762743 10.1038/s41418-021-00767-2 PMC8329302

[38] Lan YY , Heather JM , Eisenhaure T , et al. Extranuclear DNA accumulates in aged cells and contributes to senescence and inflammation. Aging Cell. 2019;18(2):e12901. 30706626 10.1111/acel.12901 PMC6413746

[39] Tan X , Tao Q , Li G , et al. Fibroblast growth factor 2 attenuates renal ischemia‐reperfusion injury via inhibition of endoplasmic reticulum stress. Front Cell Dev Biol. 2020;8:147. 32266254 10.3389/fcell.2020.00147 PMC7105877

[40] Fouillet A , Levet C , Virgone A , et al. ER stress inhibits neuronal death by promoting autophagy. Autophagy. 2012;8(6):915‐926. 22660271 10.4161/auto.19716 PMC3427257

[41] Tabas I , Ron D . Integrating the mechanisms of apoptosis induced by endoplasmic reticulum stress. Nat Cell Biol. 2011;13(3):184‐190. 21364565 10.1038/ncb0311-184 PMC3107571

[42] Zhang D , Liu Y , Zhu Y , et al. A non‐canonical cGAS–STING–PERK pathway facilitates the translational program critical for senescence and organ fibrosis. Nat Cell Biol. 2022;24(5):766‐782. 35501370 10.1038/s41556-022-00894-z

[43] Steiner A , Hrovat‐Schaale K , Prigione I , et al. Deficiency in coatomer complex I causes aberrant activation of STING signalling. Nat Commun. 2022;13(1):2321. 35484149 10.1038/s41467-022-29946-6 PMC9051092

[44] Radford H , Moreno JA , Verity N , Halliday M , Mallucci GR . PERK inhibition prevents tau‐mediated neurodegeneration in a mouse model of frontotemporal dementia. Acta Neuropathol. 2015;130(5):633‐642. 26450683 10.1007/s00401-015-1487-z PMC4612323

