# Supplementary figures and images for: PERK‐STING‐RIPK3 pathway facilitates cognitive impairment by inducing neuronal necroptosis in sepsis‐associated encephalopathy
Source: CNS Neurosci Ther. 2023 Jan 24;29(4):1178–91. doi: 10.1111/cns.14095 (PMC10018099; doi:10.1111/cns.14095)

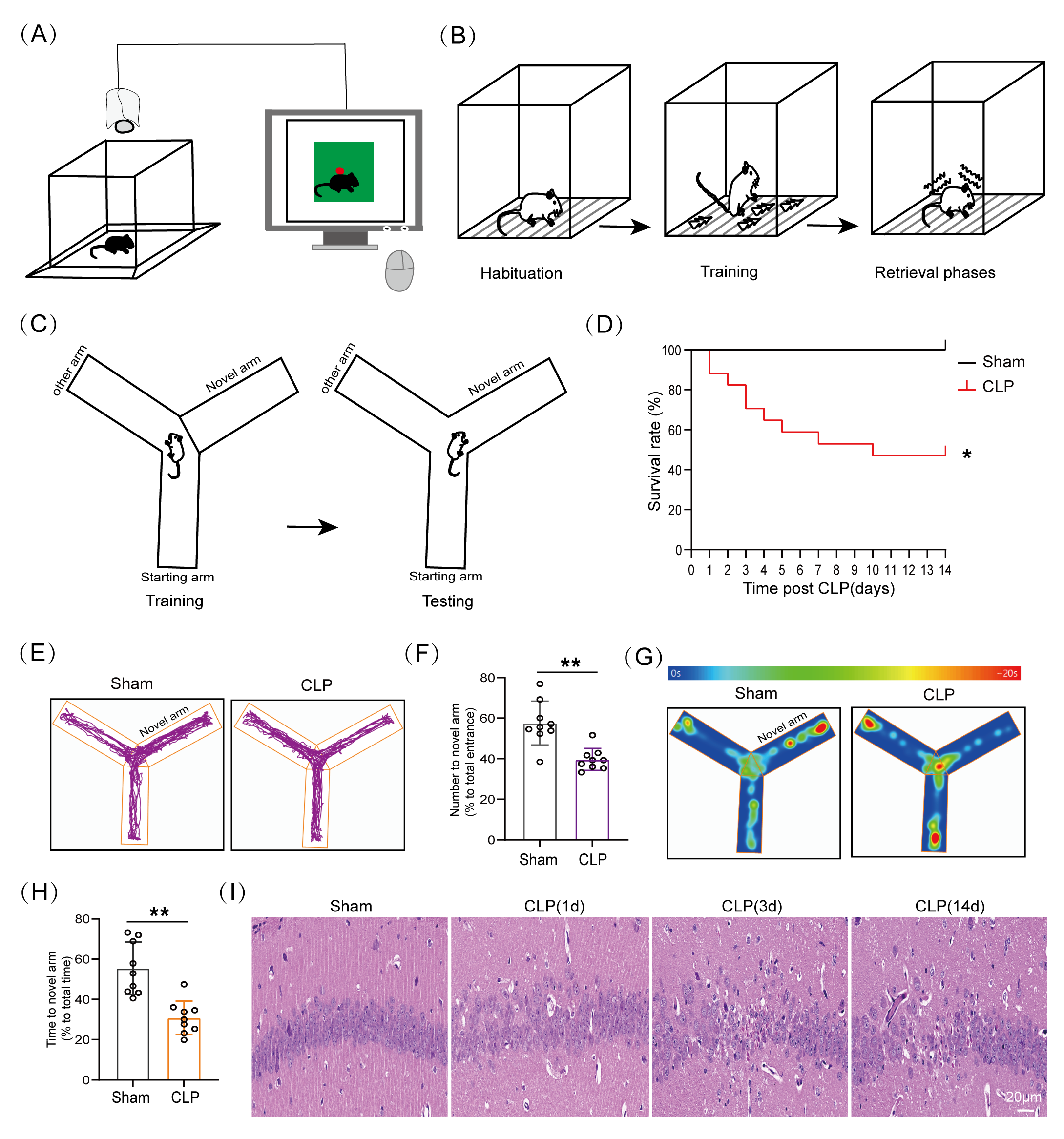

Supplement: Supplementary file 1 — Figure S1 [file CNS-29-1178-s004.tif]

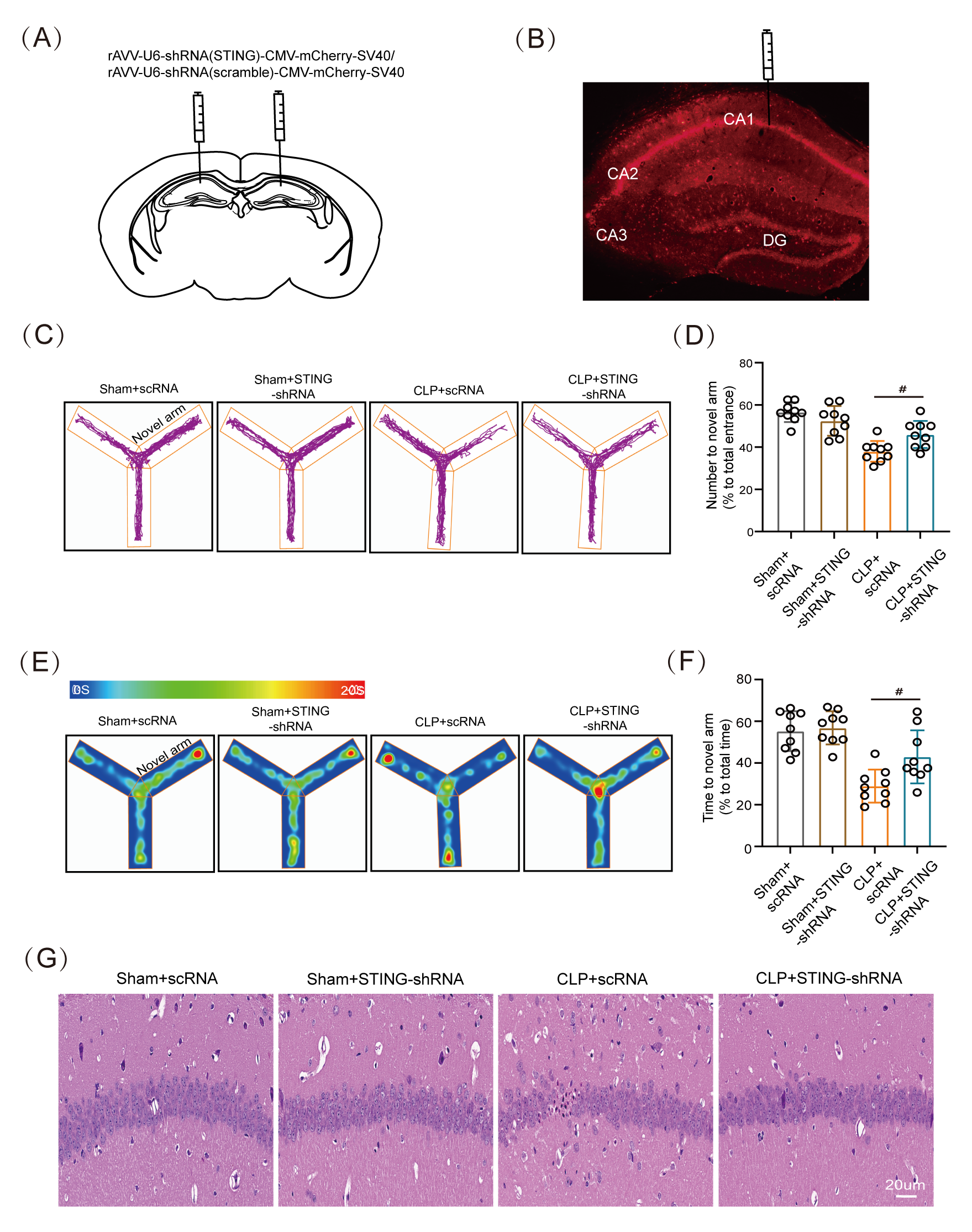

Supplement: Supplementary file 2 — Figure S2 [file CNS-29-1178-s001.tif]

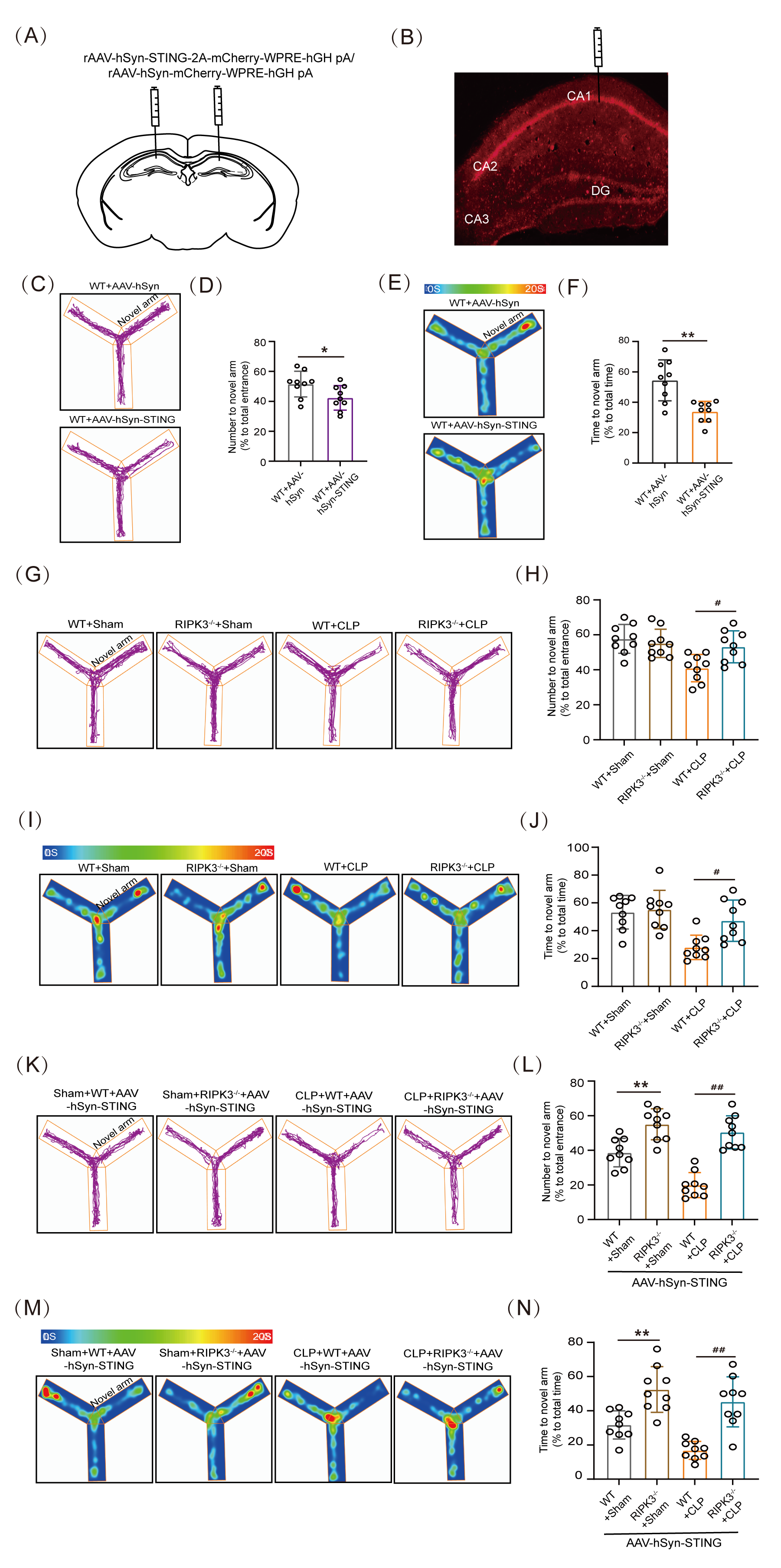

Supplement: Supplementary file 3 — Figure S3 [file CNS-29-1178-s002.tif]

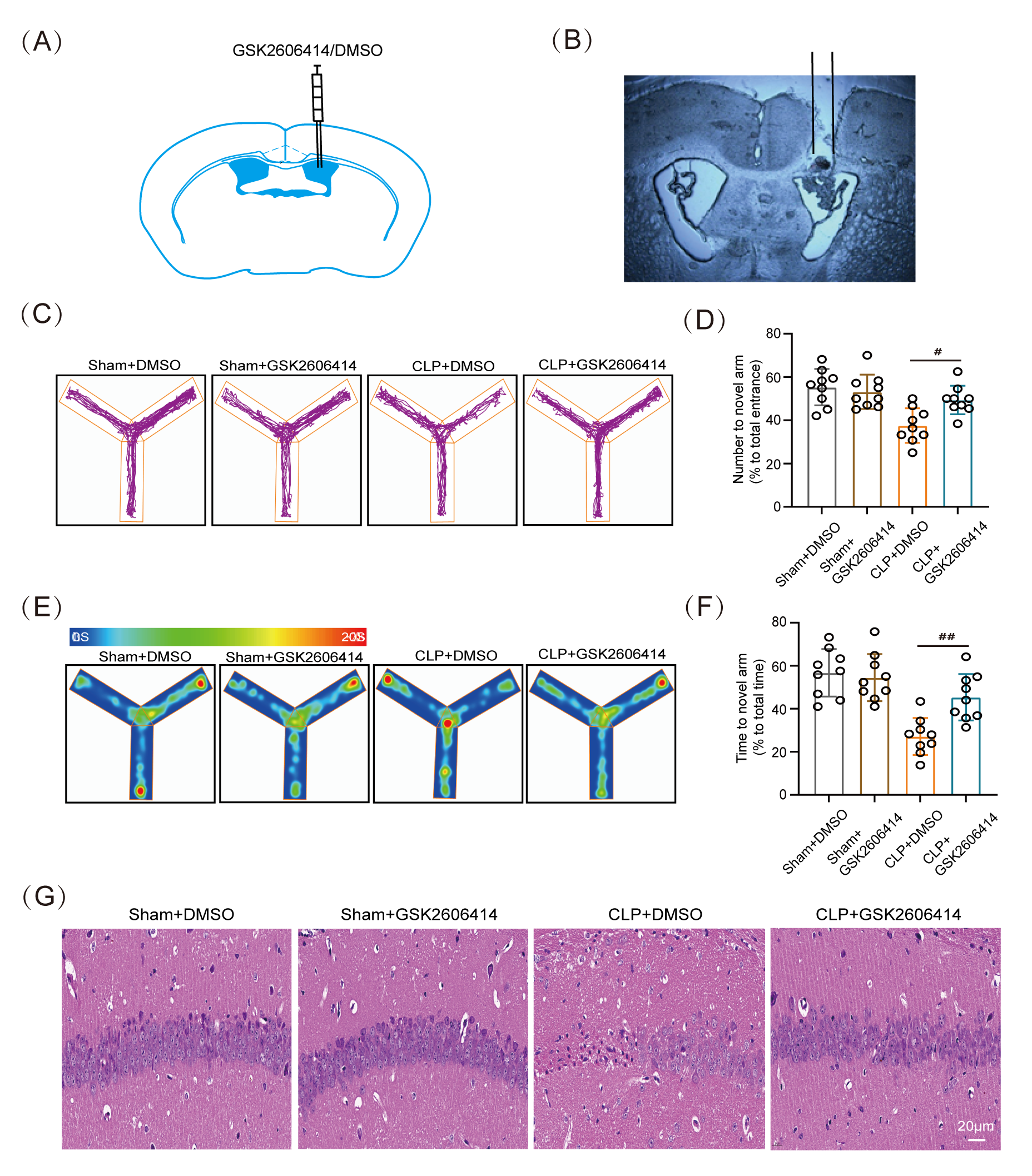

Supplement: Supplementary file 4 — Figure S4 [file CNS-29-1178-s003.tif]
